# Development and evaluation of a soft wearable weight support device for reducing muscle fatigue on shoulder

**DOI:** 10.1371/journal.pone.0173730

**Published:** 2017-03-14

**Authors:** Daegeun Park, Kyu-Jin Cho

**Affiliations:** School of Mechanical and Aerospace Engineering/Institute of Advanced Machines and Design, Seoul National University, Seoul, Republic of Korea; Shanghai Jiao Tong University, CHINA

## Abstract

Compensating the weight of human limbs is important in reducing muscle fatigue experienced by manual laborers. In this study, a compact and lightweight soft wearable weight support device was developed and evaluated. The device supports gravitational force on the shoulder at any arm posture, although there are some limitations in its assistive performance. The device actuator consists of a cam-rod structure, a tendon-driven mechanism, and a rubber band. The desired assistive torque is translated to the shoulder joint along a tendon routing structure. Device performance was evaluated by measuring muscle activation in with-assist and without-assist conditions. Muscle activation on the deltoid was measured by surface electromyography. An experimental protocol consisting of a series of exercises was executed with six healthy subjects. The subjects raised and lowered their arm from 0 to 100 degrees for 30 times under eight conditions, which were combined with-assist and without-assist conditions, and holding the horizontal angle of the arm at 0, 30, 60, or 90 degrees against the sagittal plane. Surface electromyography data were pre-processed and analyzed using a root mean square method. When muscle fatigue occurs, the root mean square of the surface electromyography increases nonlinearly. This was calculated using the standard deviation of the root mean square. Three of six subjects showed decreased variation of the root mean square between the exercises in the with-assist condition. One subject’s result was significantly reduced (by about 57.6%) in the with-assist condition. In contrast, two subjects did not show significant difference between measurements taken in the with-assist and without-assist conditions. One subject was dropped from the experiment because the device did not fit the subject’s body. In conclusion, the effectiveness of the soft wearable weight support device in supporting shoulder movements was verified through the decreased variation of muscle activation.

## Introduction

Reducing muscle fatigue that manual laborers can experience during work is important because fatigue can destabilize the body’s ability to sense and control joint position [1], eventuating in overworked muscles. Repeated bouts of muscle overwork lead to cumulative trauma that can cause muscle strain injuries and bone strain [2,3], especially in industries that demand repetitive work tasks [4–6]. Such musculoskeletal damage decreases laborers’ ability to work and reduces their overall working life expectancy. Musculoskeletal damage to the arm is of particular significance because it seriously complicates not only manual work tasks but also the activities of daily living (ADLs). To safeguard laborer’s musculoskeletal health, working life expectancy, and overall quality of life, effective solutions for reducing muscle fatigue of the arms during repetitive work tasks are required.

Many assistive devices have been developed to reduce muscle fatigue by supporting the movement of the arms and other parts of the musculoskeletal system. Simple limb support devices [7,8] are generally attached to a body limb and reduce muscle fatigue by dispersing loads or pressure applied to joints and muscles. These devices tighten the joint structurally by increasing its stiffness. They do not provide any additional force to directly assist limb movement. To a degree, this approach can stabilize limb movement and reduce the burden on joints and muscles. However, these devices can also cause discomfort when the targeted limb is moved.

Several active assistive devices that generate force to assist limb movement have been developed, such as HAL [9–11], eLeg [12,13], REX [14], and ReWalk [15]. These devices use a variety of approaches [16–18] to apply force in the desired direction to reduce the burden on muscles during work. Generally, they consist of active motors with a complex linkage structure, sensors, and a control system. These devices detect a user’s intention via sensors and push or pull limbs actively using their motors and linkage structure to apply large directional assistive forces. Complicated algorithms such as impedance control are used to control the whole system. Active assistive devices augment human movement by providing higher degrees of freedom (DOF) to limbs and allowing them to exert more power than they can naturally.

In contrast to active assistive devices, passive assistive devices apply less power to limbs. Several passive assistive devices, such as the Armon Edero [19], WREX [20], and others [21,22], use a rubber band or a spring as a passive actuator and do not provide any active control. These devices support the arms against gravity. The fundamental principle of all passive assistive devices is energy conservation. Assistive force is generated by storing energy in passive actuators and using passive height change of the arm to convert the stored energy from potential to active energy. Although these devices deliver limited additional DOF and assistive power compared to active assistive devices, they have wide appeal owing to their simple design and ease of use.

Despite the effectiveness of active and passive assistive devices, several challenges remain to be overcome for them to be useful to manual laborers. The most important challenge is reducing device size. Traditionally, wearable devices are made of rigid materials to facilitate the application of assistive force to the limbs in the desired direction. Devices designed with this approach require complicated and bulky structures [23–26] as a result of trying to assist complex human joints (roll-and-glide, saddle, ball-and-socket, etc.) [27]. Additional structures are also required, to mount the actuators on the limbs, which further increases device size. These bulky structures in turn require a larger work space, which ultimately limits their use because it is usually impossible to increase available work space.

To overcome these challenges, our wearable weight support device combines soft robotics and a tendon-driven mechanism. Soft robotics concepts [28] have been applied to create numerous soft wearable assistive devices such as our device. Soft robots have multiple DOF regardless of structure, which makes it is easier to give them complex joint movements. Although this makes it difficult to constrain unwanted DOF in soft wearable assistive devices, this is resolved by using human joints as the devices’ robotic frame. This approach makes it possible to reduce structural size and complexity while maintaining structural strength [29–32]. Adding a tendon-driven mechanism can help reduce the burden caused by device size. This mechanism allows the device’s actuator to be located at any body part because assistive force is transmitted through the tendon, which helps minimize device size and reduce obstacles in body movement.

In this paper, we develop and evaluate a soft wearable weight support device based on a tendon-driven mechanism that assists shoulder torque in the gravitational direction. To ensure freedom of movement, the device compensates for the torque generated by the weight of the arm, because this is the main disturbed force among various directional forces. We suggest two compensatory mechanisms to solve several issues related to force transmission that are inherent in the soft robot concept and tendon-driven mechanisms.

Force transmission is different in our soft wearable weight support device compared to a rigid wearable device. A rigid wearable device applies normal force to the target limb via its rigid linkage structure, which rotates the limb. In contrast, our device applies force to the target limb by pulling its tendon, which applies both shear force and normal force. The shear force must be minimized because it is of no use in rotating the limb. It only serves to compress the joint uncomfortably and cause the device to rub against the skin, which can be painful. To reduce shear force, our device employs a passive actuation system consisting of a noncircular cam structure and uses a rubber band as a power source [33,34]. This arrangement reduces shear force by generating a sinusoidal assistive force profile, which is similar to the profile of the force exerted when the arm weight follows the arm angle.

To generate assistive torque against gravity, the actuation tendon should always pull the upper arm in an upward direction. However, if the tendon is attached to a fixed position on the upper arm, the pulling direction will change as the upper arm moves, which will apply the assistive force transmitted through the tendon in an undesired direction. To resolve this issue, we used a hooked tendon routing mechanism to maintain the actuation tendon routing in an upward direction at any shoulder posture.

To evaluate the proposed soft wearable weight support device, muscle activation was measured by monitoring surface electromyography (sEMG) signals during repeated shoulder exercises in various postures, because muscle fatigue is indicated through a nonlinear increase in muscle activation [35]. The nonlinearity of the muscle activation is calculated through the standard deviation (SD) of the root mean square (RMS) of sEMG for each exercise. Although the assistive force did not fully support the torque caused by arm weight, muscle fatigue was significantly reduced throughout the movements. This proves that this soft wearable weight support device can reduce muscle fatigue during repetitive motions, and is significantly effective as an assistive device in actual work situations.

## Methods

### Design of the soft wearable weight support device

**Fig 1:**
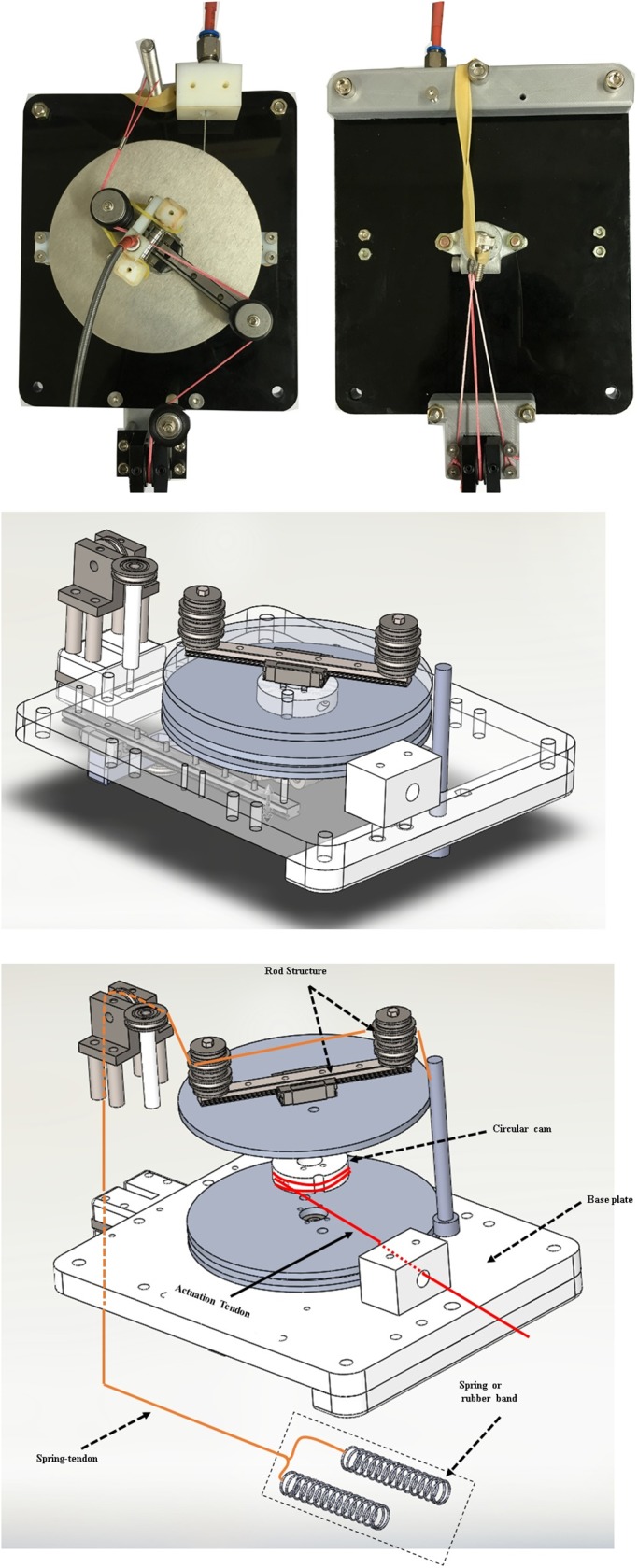
Passive actuation system. The passive actuation system consists of the cam-rod structure, base plate, spring-tendon, and a rubber band.

**Fig 2:**
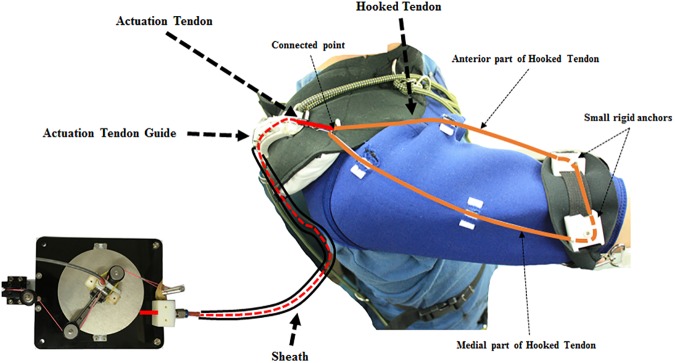
Tendon routing structure. The tendon routing structure consists of an actuation tendon, a hooked tendon, an actuation tendon guide, and a sheath.

**Fig 3:**
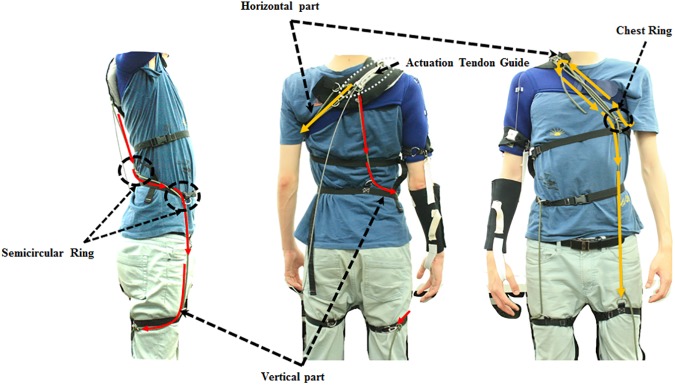
Anchoring structure. The anchoring structure consists of the vertical part and the horizontal part.

**Fig 4:**
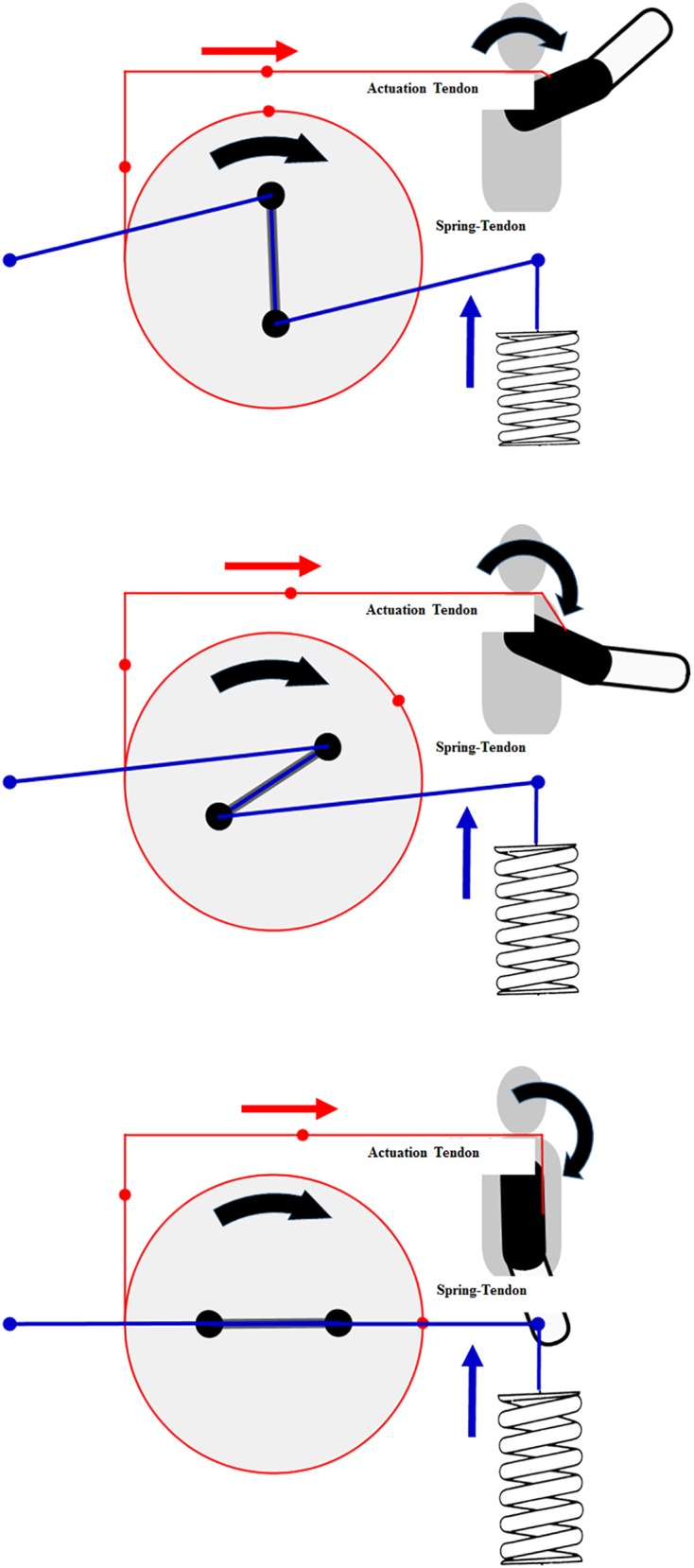
Schematic of the actuation system operation.

**Fig 5:**
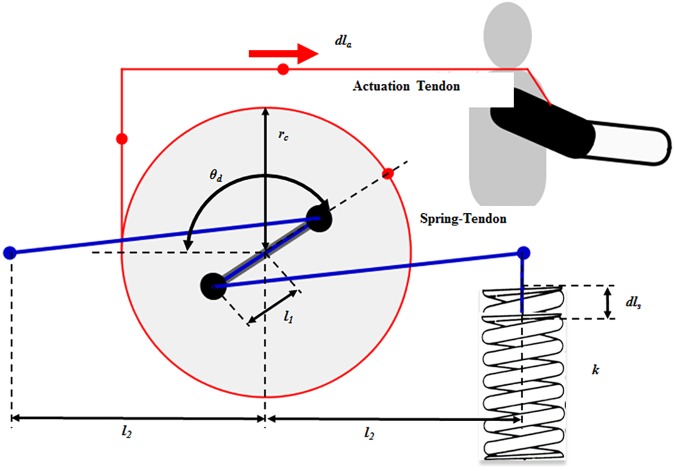
Actuation system parameters.

The soft wearable weight support device was designed to support the torque on the shoulder caused by arm weight. It consists of three main elements: a passive actuation system (Fig 1), a tendon routing structure (Fig 2), and an anchoring structure (Fig 3). The actuation system was designed using previously investigated concepts [33,34]. Its purpose is to reduce shear force on the limb, which only compress the shoulder joint and causes the device to rub painfully against the skin, without helping to assist the shoulder against gravity. The actuation system consists of a cam-rod structure, a spring-tendon, a rubber band, and a base plate. The cam-rod structure consists of a rod structure with pulleys and a circular cam. The rod structure is attached to the circular cam, and the cam-rod structure is linked to the base plate. The cam-rod structure can be rotated on the base plate owing to bearings between these two components. The spring-tendon attaches the base plate to the rubber band beneath the base plate by wrapping the pulley on the rod structure. When the cam-rod structure is rotated, the spring-tendon is pulled or released, which elongates or loosens the rubber band. Since the tendon routing structure attaches the circular cam to the upper arm, the actuation system is operated by the upper arm movement. This generates elastic force on the rubber band, which is transmitted to the upper arm through the tendon routing structure. Finally, the elastic force applies an assistive force to the shoulder to support the shoulder torque against gravity (Fig 4). It is derived by the change of the actuation tendon length, as shown in Eqs 1, 2 and 3. The equation parameters are defined in Fig 5.

$$\theta_{d}=\frac{{dl}_{a}}{r_{c}}$$ (Eq 1)

$${dl}_{s}=2\sqrt{{\left(l_{2}-l_{1}\cos\theta_{d}\right)}^{2}+{\left(l_{1}\sin\theta_{d}\right)}^{2}}+2l_{1}-l_{s,0}$$ (Eq 2)

$$F_{elastic}=k\times {dl}_{s}=k\times \left(2\sqrt{{\left(l_{2}-l_{1}\cos\frac{{dl}_{a}}{r_{c}}\right)}^{2}+{\left(l_{1}\sin\frac{{dl}_{a}}{r_{c}}\right)}^{2}}+2l_{1}-l_{s,0}\right)$$ (Eq 3)

In Eq 1, *θ*_*d*_ is the angle of the rod structure, *dl*_*a*_ is the pulled or released length of the actuation tendon, and *r*_*c*_ is the radius of the circular cam. In Eq 2, *l*_1_ is the half length of the rod structure, *l*_2_ is the length between the center of rotation of the circular cam and the attached position of the spring-tendon, *l*_*s*,0_ is the initial length of the spring-tendon when *θ*_*d*_ is zero, and *dl*_*s*_ is the pulled or released length of the spring-tendon. In Eq 3, k is the spring coefficient of the spring, and *F*_*elastic*_ is the elastic force in the rubber band.

To transmit the assistive force to the upper arm, the tendon routing structure consists of an actuation tendon and a hooked tendon (Fig 2). The actuation tendon connects the circular cam in the actuation system and the hooked tendon. One end of the actuation tendon is attached to the circular cam by being wrapped around the cam, and the other end passes through the tendon sheath and attaches to the hooked tendon. One end of the sheath is attached to the base plate and the other end to an actuation tendon guide located on the shoulder. The actuation tendon exits the sheath at the actuation tendon guide, and connects to the hooked tendon exiting the sheath. Because the sheath is incompressible and its length cannot change, the actuation tendon inside the sheath also cannot change length, and thus the actuation system can be located anywhere without affecting actuation tendon length. The change of the actuation tendon length is defined as the distance between the actuation tendon guide on the shoulder and the hooked tendon on the upper arm. Therefore, when the upper arm is moved, the actuation tendon is pulled or released, which generates an elastic force that acts as an assistive force. As a result, the total amount of applied assistive force is derived from the upper arm movement. By simplifying the shoulder shape as a sphere, the pulled length of the actuation tendon is derived from Eq 4, and the relationship between the upper arm angle and the assistive force and torque is derived from Eqs 5, 6 and 7.

$$|d_{1}|=r\times \theta_{u}={dl}_{a}=r_{c}\times \theta_{d}$$ (Eq 4)

$$\tau_{c}=F_{elastic}\times (\frac{l_{2}\times l_{1}\sin\theta_{d}}{sqrt(l_{1}^{2}+l_{2}^{2}-2l_{1}l_{2}\cos\theta_{d})})$$ (Eq 5)

$$F_{assist}=\frac{\tau_{c}}{r_{c}}$$ (Eq 6)

$$\tau_{a}=r\times F_{assist}$$ (Eq 7)

**Fig 6:**
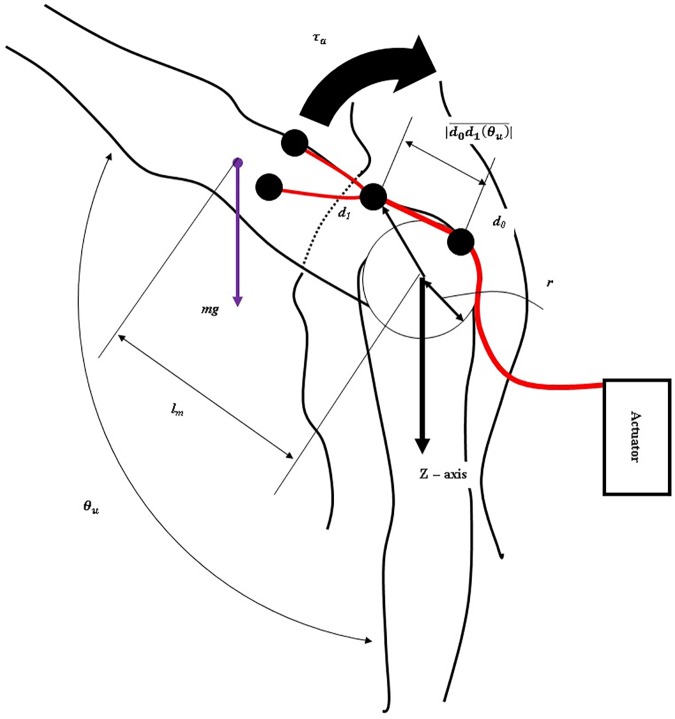
Tendon routing structure and human body parameters.

In Eq 4, *d*_1_ is the position of the end of the actuation tendon that is attached to the hooked tendon, *r* is the radius of the shoulder shape, and *θ*_*u*_ is the angle between the upper arm and the z axis on the shoulder joint (Fig 6). |*d*_1_| is the pulled length of the actuation tendon. In Eq 5, *τ*_*c*_ is the torque on the cam-rod structure. Eq 6 derives the relationship between the torque on the cam-rod structure (*τ*_*c*_) and the assistive force (*F*_*assist*_). Eq 7 derives the assistive torque (τ_*a*_) which is applied on the shoulder in order to support the shoulder torque caused by the arm weight against gravity. The weight of the total arm is assumed to be 3 kg, and the profile of the actual required torque is assumed to have a sinusoidal shape. Parameter values for the assistive torque (Table 1) were chosen to ensure that the maximum assistive torque is the same as the maximum required torque. Eq 8 derives the actual required torque in the static state.

$$\tau_{required}=mgl_{m}\;\sin\theta_{u}$$ (Eq 8)

**Table 1:** Parameter values to simulate the assistive torque and the actual required torque.

	Parameters	Value
**Actuation system**	*l*_*1*_	0.045 m
	*l*_*2*_	0.072 m
	*r*_*c*_	0.05 m
	*k*	940 N/m
**Human Body**	*r*	0.05 m
	*d*_*1*_	0.1 m
	*m*	2.86 kg
	*l*_*m*_	0.3 m

**Fig 7:**
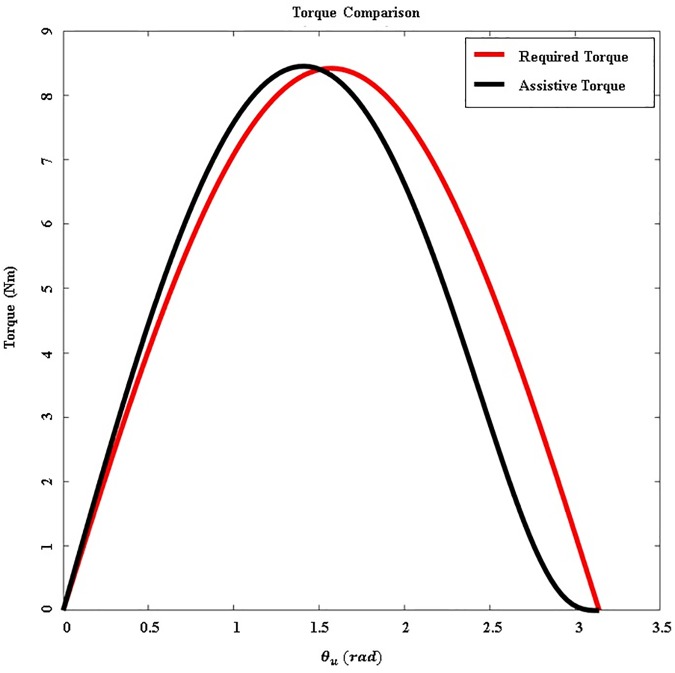
Comparison between assistive torque and the actual required torque. The red line indicates the actual required torque necessary to support the weight of the arm. The black line indicates the assistive torque generated by the actuation system.

In Eq 8, *m* is the total mass of the arm, *g* is the gravitational acceleration, and *l*_*m*_ is the moment arm of the whole arm. Fig 7 compares the amount of assistive torque and actual required torque. This figure shows a calculation of the actual torque required to raise the upper arm with a fully extended elbow. The difference between the assistive torque and the actual required torque is large, between 100 and 180 degrees. However, this actuation system is effective enough because the upper arm typically moves under 100 degrees during ADLs [24,36,37].

**Fig 8:**
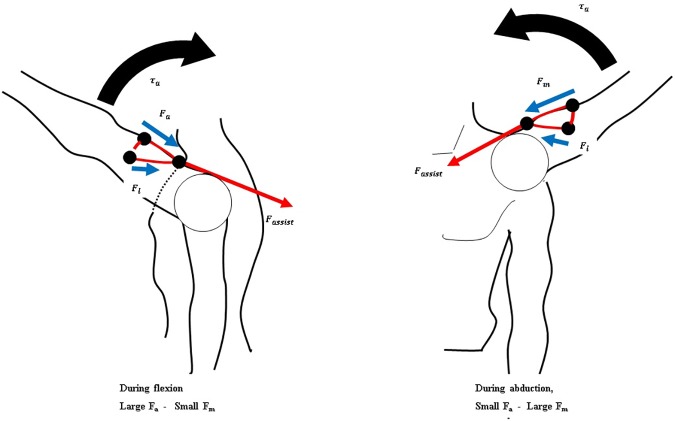
Schematic of the force distribution applied to the hooked tendon according to arm posture.

To apply the assistive force against gravity, the direction of the force on the upper arm must be upwards. Also, because the force required to assist the shoulder depends on vertical movement of the arm, horizontal arm movement must be decoupled from the generation of the assistive force (Fig 6). The hooked tendon is designed for two purposes: to change the direction of the assistive force to an upward direction and to decouple the generation of assistive force from horizontal arm movement. The hooked tendon is connected to the actuation tendon at a connected point over the center of rotation of the shoulder joint and routed around two small rigid anchors on the upper arm. One rigid anchor is attached to the anterior part of the upper arm, and the other is attached to the medial part of the upper arm (Fig 2). Through these, the assistive force of the device is distributed on the anterior and the medial parts of the upper arm. The length of both parts of the hooked tendon changes in response to changes in upper arm posture, which in turn changes the direction of the actuation tendon. Thus, assistive force is distributed to both parts of the hooked tendon, which pulls the upper arm in the upward direction at the ventral side (Fig 8). The distributed forces may be derived with Eqs 9 and 10. Because the arms generally move ventrally during manual labor [24,36,37], this tendon routing structure is designed to assist motion at the ventral side.

$$\vec{F_{m}}=mgsin\theta_{v}=\vec{F_{a}}+\vec{F_{l}}=\vec{F_{}}$$ (Eq 9)

$$\vec{F_{a}}=\vec{F_{m}}cos\theta_{h},\;\vec{F_{l}}=\vec{F_{m}}sin\theta_{h}$$ (Eq 10)

**Fig 9:**
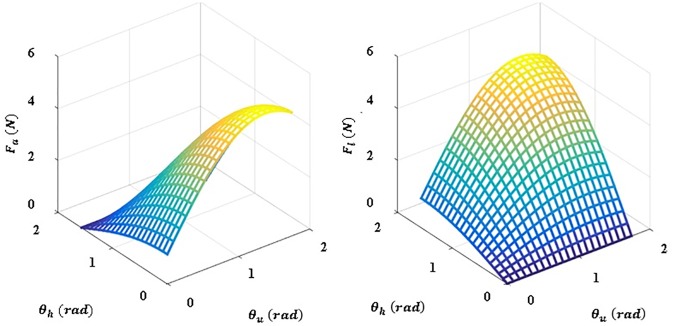
Simulation depicting the force distribution applied to the hooked tendon. F_a_ is defined as the force applied to the anterior part of the hooked tendon and F_l_ is defined as the force applied to the medial part of the hooked tendon.

Eqs 9 and 10 show the derivation of the force distribution on the hooked tendon. *F*_*m*_ is the force generated by the arm mass when the arm is raised, *F*_*a*_ is the force transmitted in the anterior direction on the upper arm, *F*_*l*_ is the force transmitted in the lateral direction on the upper arm, and *F* is the actuated force from the actuation. The force distribution is shown in Fig 9. This figure shows that the direction of actuated force is maintained by the hooked tendon routing to pull the upper arm against gravity.

**Fig 10:**
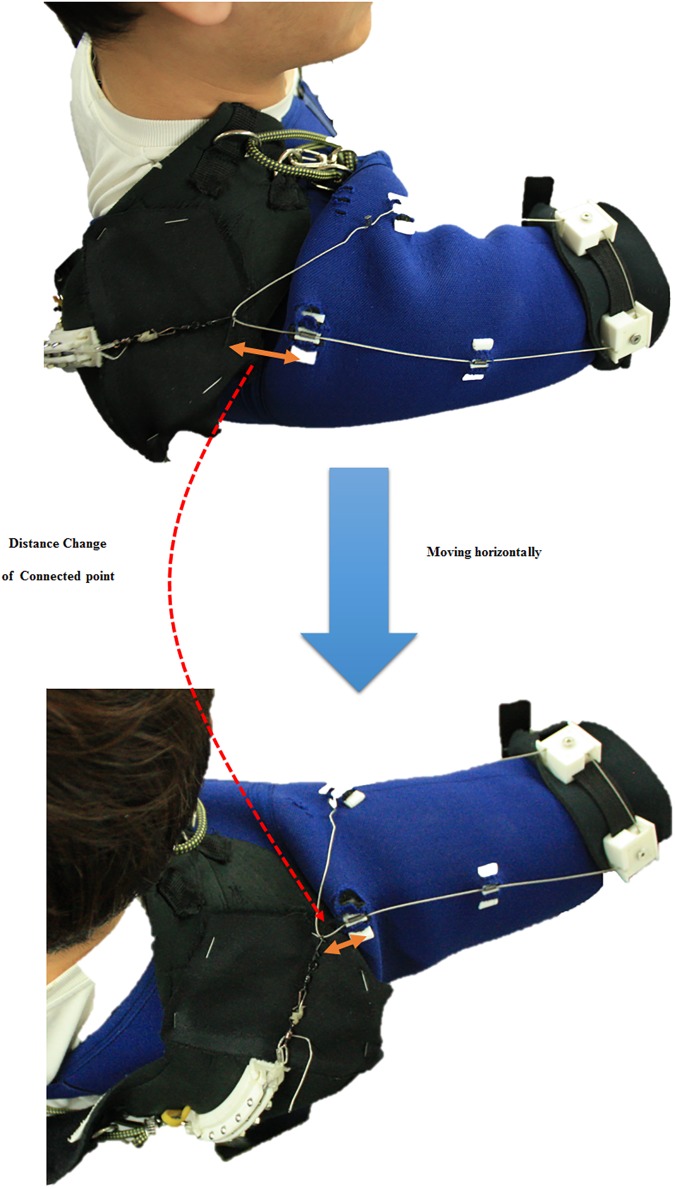
Distance change with respect to upper arm motion on the connected point of the hooked tendon. Orange arrows indicate the distance between the connected point of the hooked tendon and the actuation tendon guide.

**Fig 11:**
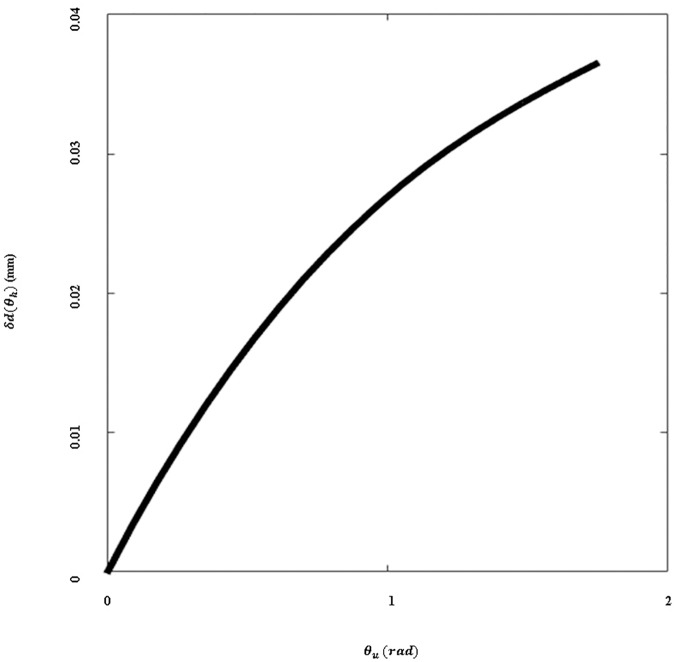
Elongation of the actuation tendon with respect to distance change of the connected point. The change in elongation when the subject moves their arm in the horizontal plane at any given vertical angle.

To decouple changes in the amount of assistive force from horizontal arm movement, the hooked tendon moves freely from the anterior part to the medial part (Fig 10). Owing to this structure, the length of the actuation tendon changes minimally even if the subject moves the arm in a horizontal direction at the same height, as per Fig 11. There is little length change on the hooked tendon, and any change that occurs can be compensated for by deformation of the soft wearable parts.

$$\vec{d_{i}}=R_{h}R_{u}\vec{d_{i}^{0}}\;(i=2,3)$$ (Eq 11)

$$\delta d(\theta_{h})=\bar{d_{0}d_{1}(\theta_{h})}-\bar{d_{0}d_{1}(\theta_{h,0})}$$ (Eq 12)

**Fig 12:**
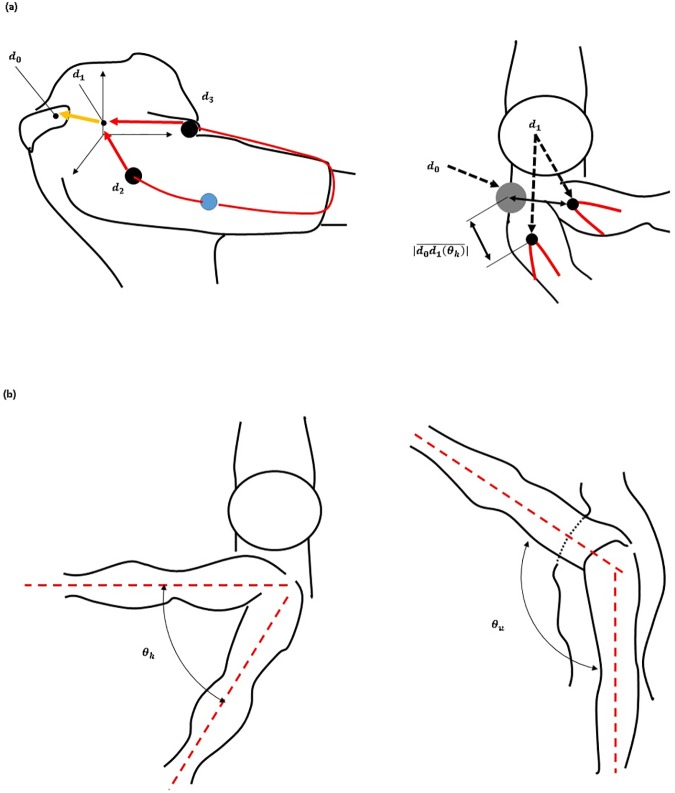
Hooked tendon parameters.

In Eqs 11 and 12, *θ*_*h*_ is the angle between the sagittal plane on the shoulder and the upper arm, *d*_*i*_ (*i* = 2,3) are the positions of the attachment points on the hooked tendon, *R*_*h*_ and *R*_*u*_ are the rotational matrices of each angle (*θ*_*h*_ or *θ*_*u*_), and *δd*(*θ*_*h*_) is the change in the length of the hooked tendon when the arm moves in the horizontal plane. The parameters are defined in Fig 12.

To reduce deformation of the tendon routing structure caused by shear force, it is important to anchor structure in position. Deformation of tendon routing structure is an especially critical issue in this soft wearable weight support device because the assistive torque is determined by the geometry of the tendon routing structure. The device uses an anchoring structure which consists of a vertical anchoring part and a horizontal anchoring part. The vertical anchoring part links the actuation tendon guide to the same-side thigh. The path of the vertical anchor rope curves at the waist from the back to the abdomen by passing through semicircular rings on a waist belt and then descends to the thigh. The vertical anchoring part allows upward force generated on the actuation tendon guide to be distributed on the thigh and the waist. The horizontal anchoring part is attached to the thigh opposite from the vertical anchoring part via two ropes. One rope wraps the user’s chest and passes through a chest ring that prevents the rope from pressing up into the armpit. The other passes over the shoulder from the actuation tendon guide, passes through the chest ring, and attaches to the thigh that is opposite from the vertical anchoring part. The horizontal anchoring part ensures that the actuation tendon guide is fixed in the correct position when it receives horizontal force.

### Experimental protocol

An experimental protocol was designed to evaluate the performance of the soft wearable weight support device and approved by the Seoul National University Institutional Review Board (SNUIRB). All subjects provided written consent to participate in this study and this consent procedure was approved by SNUIRB. Six right-handed healthy subjects (five males, one female, aged 25.3 ± 1.5 years; weight 58.2 ± 6.8 kg; height 167 ± 6.2 cm) participated in the protocol. Unfortunately, one subject was dropped because the assistive device did not fit the subject’s body. The remaining subjects did not have any skeletal or muscular diseases.

**Fig 13:**
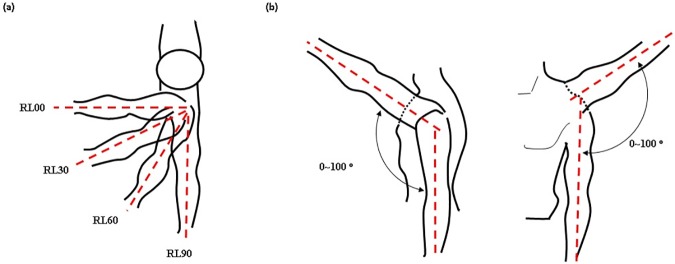
Experimental motion diagrams. (a) Top-down transverse plane view of arm posture for the RL00, RL30, RL60, and RL90 motions. (b) Range of motion between 0 and 100 degrees.

**Fig 14:**
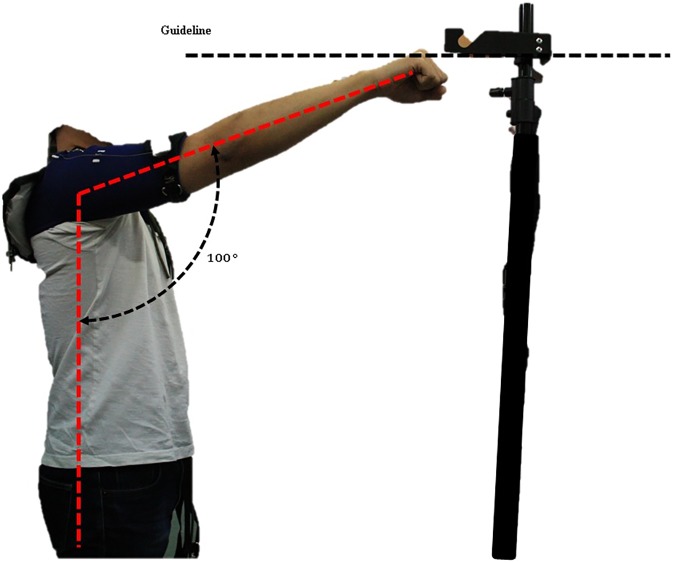
Upper arm motion range indication guideline. Adjustable guideline height according to subject height.

The protocol was scheduled to take six weeks, to provide time for subjects to recover from muscle fatigue. The protocol involved performing eight exercises that combined four motions and two conditions. The device was applied to the subjects at the right shoulder, and the subjects raised and lowered the right arm from 0 to 100 degrees keeping the horizontal angle of the arm at 0(RL00), 30(RL30), 60(RL60), or 90(RL90) degrees against the sagittal plane (Fig 13). These motions were designed to evaluate the effect of the hooked tendon mechanism at the ventral side. RL00 was designed to evaluate the device’s effect on anterior movement of the arm because the anterior part of the hooked tendon strongly pulled the upper arm in the flexion direction at RL00. RL90 was designed to evaluate the device’s effect on lateral movement of the arm, because the medial part of the hooked tendon strongly pulled the upper arm in the abduction direction. RL30 and RL60 were designed to evaluate the device’s effect on both directional movements of the arm. At these motions, the anterior and medial parts of the hooked tendon worked together to pull the upper arm vertically keeping the horizontal angle of the arm at 30 or 60 degrees against the sagittal plane. The subjects executed each motion for two seconds at a rate of about 100 degrees/sec. A 1-Hz beep was used to tell subjects when to raise or lower their arm. To ensure that subjects moved their arm to the correct angle, they were shown an indicator that guided the arm angle (Fig 14). The height of the guideline in Fig 14 is adjustable as per subjects’ height. Each motion was performed under two conditions: with assistance (WA) and without assistance (WO). During all exercises, the subjects grasped a 2-kg weight in their hand. Between exercises, the subjects rested for 5–10 minutes to recover muscle strength. A similar experimental protocol was executed in [38].

During the first week, the subjects executed the RL00 and RL90 motions under the WA and WO conditions to familiarize themselves with the experimental protocol. The data in week one were not used to analyze muscle fatigue. Six subjects executed a set of motion/condition exercises 20–25 times. Each set consisted of the following sequence of exercises: RL00-WO, RL00-WA, RL90-WO, and RL90-WA. One subject was dropped from this trial. For the remaining five weeks, five subjects executed eight exercises that combined four motions and two conditions. Table 2 shows the exercise schedule. Each motion was repeated 30 times. The number of repetitions was set accordingly to the subjects’ opinion about the level of arm muscle fatigue they were experiencing.

**Table 2:** Exercise schedule.

**Number of motion**	**Week 1**				**Week 2**				**Week 3**			
	**Exercise sequence**				**Exercise sequence**				**Exercise sequence**			
**Subject**	**RL00 WO**	**RL00 WA**	**RL90 WO**	**RL90 WA**	**RL00 WA**	**RL00 WO**	**RL90 WA**	**RL90 WO**	**RL00 WA**	**RL00 WO**	**RL90 WA**	**RL90 WO**
**1**	25	25	25	25	30	30	30	30	x	x	x	x
**2**	25	25	25	25	30	30	30	30	x	x	x	x
**3**	25	25	25	25	30	30	30	30	x	x	x	x
**4**	25	25	25	25	30	30	30	30	30	30	30	30
**5**	25	25	25	25	30	30	30	30	x	x	x	x
**6**	25	25	25	25	x	X	x	x	x	x	x	x
**Number of Motion**	**Week 4**				**Week 5**				**Week 6**			
	**Exercise sequence**				**Exercise sequence**				**Exercise sequence**			
**Subject**	**RL30 WO**	**RL30 WA**	**RL60 WO**	**RL60 WA**	**RL30 WA**	**RL30 WO**	**RL60 WA**	**RL60 WO**	**RL00 WO**	**RL00 WA**	**RL90 WO**	**RL90 WA**
**1**	30	30	30	30	30	30	30	30	30	30	30	30
**2**	30	30	30	30	30	30	30	30	30	30	30	30
**3**	30	30	30	30	30	30	30	30	30	30	30	30
**4**	30	30	30	30	30	30	30	30	30	30	30	30
**5**	30	30	30	30	30	30	30	30	30	30	30	30
**6**	x	x	x	x	x	x	x	x	x	x	x	x

**Fig 15:**
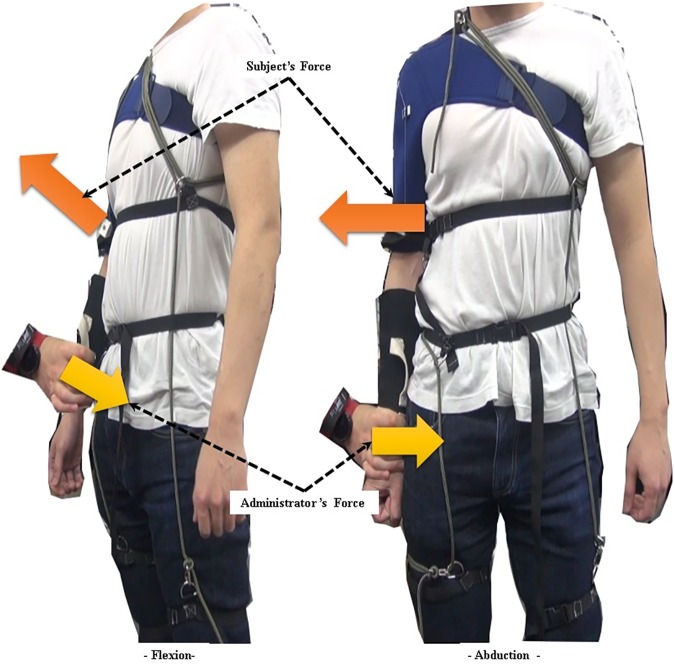
Experimental protocol. Measuring maximum voluntary contraction (MVC) in two directions, flexion and abduction.

After the subjects had donned the device but before the exercises, their maximum voluntary contraction (MVC) values were measured in two directions, flexion and abduction. To measure the MVC values, the administrator firmly held a subject’s arm at a 0 degree shoulder angle and encouraged the subject to perform shoulder flexion or abduction to their best. This process is described in Fig 15.

### Experimental setup

Muscle activation was measured with an sEMG acquisition device, Poly G-I (LAXTHA Inc., Republic of Korea). Two electrode sets were attached to the anterior deltoid (AD) and the medial deltoid (MD). These two agonistic muscles support the arm against gravity at the ventral side [39]. The ground electrode was attached to a vertebral bone on the back of the neck.

**Fig 16:**
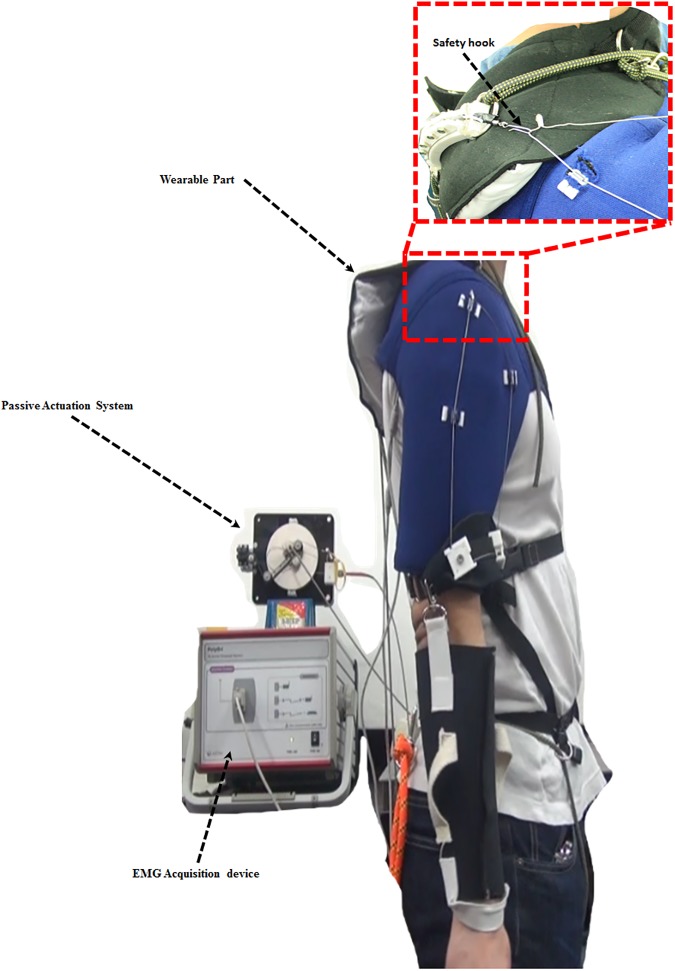
Experimental setup. Methodology for measuring deltoid sEMG signals during exercises.

The wearable part of the device consists of two elements. The first element, an arm cover, is worn at the shoulder and upper arm over the sEMG electrodes, and the hooked tendon is attached to it. The second element, the actuation tendon guide, connects to the passive actuation system and is worn over the arm cover. The subjects wore the device for all experimental exercises (Fig 16). The actuation tendon was connected to the hooked tendon for all WA exercises and disconnected from the hooked tendon for all WO exercises. The maximum assistive torque was set at about 8.6% of the maximum required torque. This percentage represents the maximum amount of assistive torque that can be applied to a subject without creating pain at the anchoring structure. The actuation tendon and the hooked tendon were connected by a safety hook, which was broken if the applied force was over 10% of the maximum required torque, to ensure the subjects’ safety (Fig 16). This safety hook is easily replaced after fracture. The maximum required torque was assumed to be the torque resulting from an arm weighing about 2.89 kg when the shoulder joint is flexed 90 degrees in the static state. The arm weight was calculated from the average of subjects’ body weight and the average percentage of the arm’s weight [40]. The device supported shoulder motion from 0 degrees to 100 degrees for all motions.

### Preprocessing and analysis of sEMG data

The raw sEMG signals were recorded at a 256-Hz sampling rate and filtered through a band-path infinite impulse response (IIR) filter with cut-off frequencies of 10 Hz and 128 Hz and a notch IIR filter with a cut-off frequency of 60 Hz to remove noise. Noise on the sEMG caused by wearing the device was negligible because the electrodes were under the same force condition in both the WO and the WA conditions. Afterward, the filtered sEMG signals were normalized for the MVC value. All analyses in the protocol were performed using a custom program written in Matlab (Mathworks Inc., USA).

**Fig 17:**
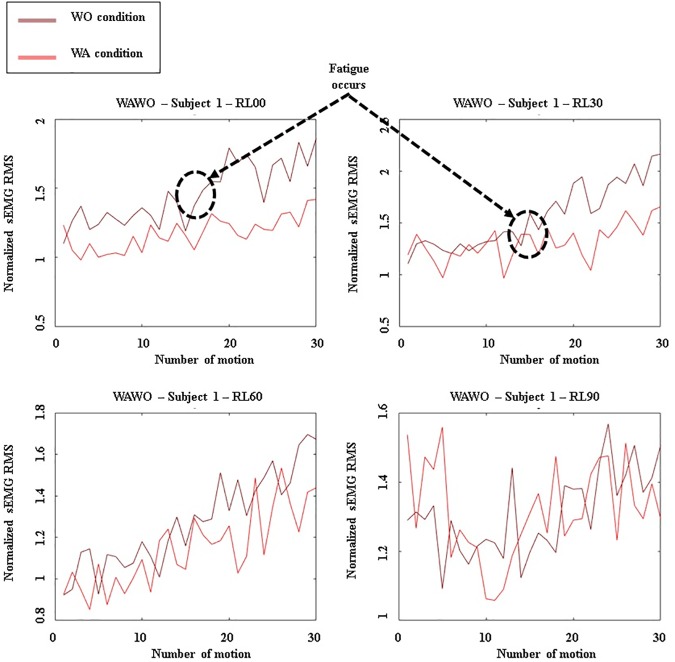
Graphs depicting the sEMG RMS AD output for subject 1. Results when WA exercises were executed before WO exercises.

The RMS values of the preprocessed sEMG data were calculated for the raising and lowering motion of each exercise. Fig 17 presents a plot of the RMS values, showing a nonlinear increase of muscle activation. Then, for each muscle, the SD of the RMS values for a given exercise was computed and normalized to given the average value of preprocessed sEMG RMS values for each exercise, and the SDs were converted to percentages. The SD values indicate the level of the nonlinear increase of muscle activation for the AD or MD muscle.

## Results

The muscle activation level changed even when subjects repeated the same motion because the sensations of muscle fatigue may have led to increasingly uneven muscle activation [41]. This uneven muscle activation indicates that the muscles were generating uneven torque since the muscle activation level increases nonlinearly [35]. In the experimental protocol, the preprocessed sEMG RMS values were compared to assess the nonlinear increase in muscle activation.

**Fig 18:**
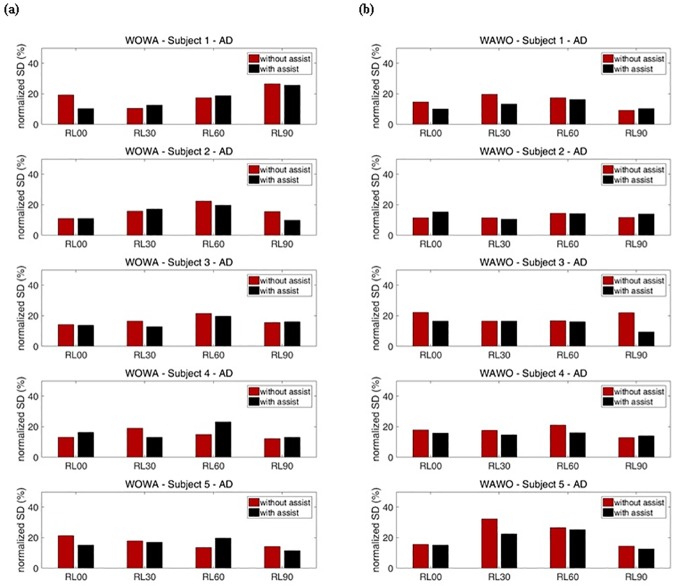
Percentage representation of with-assist (WA) and without-assist (WO) normalized standard deviations for all subjects’ AD. The red bar indicates the normalized standard deviation for without-assist exercises. The black bar indicates the normalized standard deviation for with-assist exercises. (a) Results when WO exercises were executed before WA exercises. (b) Results when WA exercises were executed before WO exercises.

**Fig 19:**
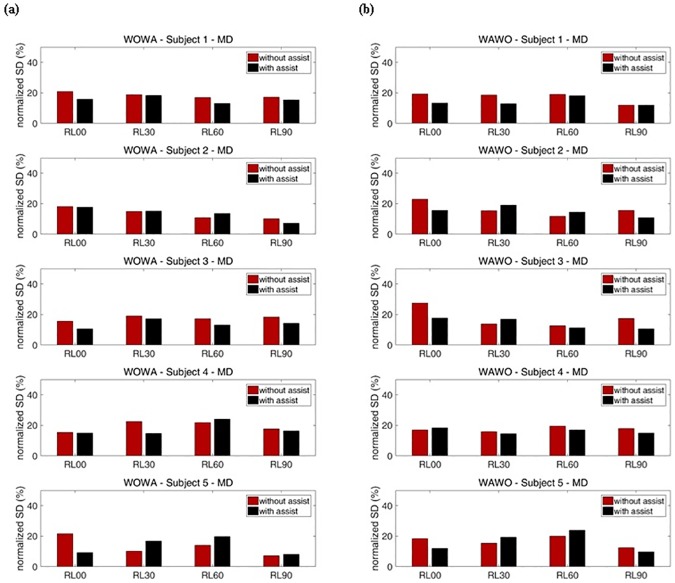
Percentage representation of with-assist (WA) and without-assist (WO) normalized standard deviations for all subjects’ MD. The red bar indicates the normalized standard deviation for without-assist exercises. The black bar indicates the normalized standard deviation for with-assist exercises. (a) Results when WO exercises were executed before WA exercises. (b) Results when WA exercises were executed before WO exercises.

Figs 18 and 19 show the SDs of the normalized RMS values for each exercise. Fig 18 shows the SDs for the AD and Fig 19 shows the SDs for the MD. To cancel out the effects of exercise order, WO exercises were executed before WA exercises on certain weeks, and WA exercises were executed before WO exercises on the other weeks (Table 2). These results show that the reduction of SDs during motion indicates that muscle fatigue decreased.

For subject 1, during RL00 and RL90 motions, the SDs for WA exercises of the AD and MD were generally smaller than the SDs for WO exercises of those muscles. During RL30 and RL60 motions, subject 1’s SDs for WO exercises were smaller than the SDs for WA exercises at the AD when WO exercises were executed before WA exercises. In contrast, subject 1’s SDs for WA exercises were smaller than the SDs for WO exercises when WA exercises were executed before WO exercises. This means that the device had little effect during RL30 and RL60 motions. At subject 1’s MD, the assistive device reduced muscle fatigue during most motions by decreasing the SDs of sEMG. For subject 2, no significant reduction of muscle fatigue was seen for either the AD or the MD. Subject 2’s SDs for WO and WA exercises were irregularly changed. For subject 3, the SDs for WA exercises were generally smaller than the SDs for WO exercises about 8.5% for the AD and 24.6% for the MD, on average. Subject 3 experienced no significant reduction of muscle fatigues in the AD during RL90 motion. Within a set, the subsequently performed WO or WA exercises showed smaller SDs. These results prove that the device had little effect during those motions. According to these results, the device significantly assisted subject 3’s arm motions for most exercises.

Subject 4 experienced no significant reduction of muscle fatigue in the AD. Moreover, when subject 4 performed RL60 motion, the SDs for WA exercises were larger than those for WO exercises by about 33.0% on average. This means that the device interrupted subject 4’s AD movements. In contrast, subject 4 experimented less MD fatigue, as indicated by about 15.7% reduction of the SDs, on average, during RL30 and RL60 motions. During RL30 motion, subject 4’s SDs for WA exercises were smaller than those for WO exercises, meaning that the device significantly assists RL30 motion. Subject 5’s results for RL00 and RL90 motions show the same tendencies subject 1’s for those motions. The average reductions of this subject’s SDs during those motions were about 23.4% for the AD and 17.6% for the MD. Subject 5 experienced maximum reduction of muscle fatigue at the AD during RL00 motion. In contrast, when subject 5 performed RL30 and RL60 motions, within a set the subsequently performed WO or WA exercises showed a larger SDs, at the MD. These results prove that the device had little effect during these motions. Overall, when half of the subjects were supported by the soft wearable weight support device, the SDs of RMS for each exercise decreased, indicating that nonlinear increases of sEMG RMS caused by muscle fatigue were reduced during the repeated motions.

## Discussion

This study was performed to extend and refine our earlier work [33,34]. Previously, we indirectly evaluated the effect of the soft wearable weight support device by measuring decreases in power consumption while the device was being used with a soft meal-assistive exoskeleton [42]. In contrast, in this study we directly evaluated the effect of the device by analyzing activation of the shoulder muscles. Moreover, in contrast to earlier versions of this device, which only supported shoulder flexion, the device used in this study was modified to support flexion/extension, abduction/adduction, and combinations of these two motions. To assess the performance of the device, we analyzed its effect on a variety of movements through various exercises including repeated motion, to more deeply investigate its performance.

The exercise protocol was designed to evaluate the unevenness of muscle activation when using the device, which was measured as SDs for sEMG RMS. Although a subject repeats the same motion, the sEMG RMS level can change as a result of the fatigue level [35,41]. Torque generated by a muscle can also change because the sEMG RMS level is nonlinearly proportional to the torque generated by muscle [35]. Accordingly, the uneven muscle activation during repeated motions indicates that muscle fatigue increased. The result shows that the unevenness of muscle activation indicated by the SDs of sEMG RMS decreased when the subject was assisted by the device. Thus, the soft wearable weight support device was able to reduce muscle fatigue during repeated motions by generating assistive torque on the shoulder joint.

The soft materials of our device constitute the main factor increasing the device’s usability in actual work situations because they enable a compact and lightweight structure. However, the device is easily deformed owing to the softness of the structure when it is under a large assistive force, especially the tendon routing structure that transmits the assistive force on the upper arm. Such deformation can cause the device to malfunction. In addition, in tests conducted prior to this study, subjects experienced some pain at the site of the anchoring structure when a large assistive force was applied. Owing to these restrictions, the assistive force in this study was not able to fully reach the level of the actual required torque caused by the arm weight. Although the low assistive torque that the device offers could meaningfully assist muscle activation, it can be made more useful by modifications that apply greater levels of assistive torque. Moreover, since the anchoring structure is separated, donning the device is complicated. To solve these challenges, we plan to improve the design of the anchoring structure by embedding rigid frames partially. Ultimately, this device can also help people with a disability if the device can set to generate enough assistive torque to overcome gravity. We will test the ability of an improved device to assist people who cannot move their arms freely because of muscular dystrophy or stroke.

In regards to the evaluation methods used in this study, measuring sEMG data on various muscles such as the pectoralis, trapezius, and teres muscle would have provided more accurate information on the effect of the device. sEMS data alone are not enough to quantitatively evaluate the quality of the assistance provided because sEMG data are hard to match with physical parameters such as force, torque, and displacement. Besides objective data, wearers’ opinions about the soft wearable weight support device are also important. It would be optimal to compare our device to traditional weight support devices in terms of physical results and wearers’ opinions. In future, we plan to employ systems to measure physical data and perform an official survey of wearers’ opinions, to evaluate the results and usability of this soft wearable weight support device more thoroughly.

## Conclusions

In this paper, a modified soft wearable weight support device with a passive actuation system was suggested and evaluated on five healthy subjects. The experimental results showed that the nonlinearity of the muscle activation for repeated motions in each muscle was improved overall although the assistive torque was a little weak. This result proves that the device can reduce muscle fatigue during repetitive motion activities. This study demonstrates the effectiveness of the soft wearable weight support device and identifies areas needing improvement.

## Supporting information

S1 Dataset MVC values for each subject at each exercise. (XLSX)

S2 Dataset Raw data of representative preprocessed sEMG RMS values for subject 1’s AD. (XLSX)

S3 Dataset Raw data of total percentage of SD values. (XLSX)
